# CSACI position statement: epinephrine auto-injectors and children < 15 kg

**DOI:** 10.1186/s13223-015-0086-9

**Published:** 2015-06-12

**Authors:** Michelle Halbrich, Douglas P. Mack, Stuart Carr, Wade Watson, Harold Kim

**Affiliations:** Paediatric Allergy, Asthma and Clinical Immunology, Winnipeg Clinic, University of Manitoba, Winnipeg, MB Canada; Department of Pediatrics, McMaster University, Pediatric Allergy, Asthma and Immunology, Assistant Clinical Professor, McMaster University, Joseph Brant Memorial Hospital, Burlington, ON Canada; Department of Pediatrics, University of Alberta, Edmonton, AB Canada; Department of Pediatrics, Dalhousie University, Head, Division of Allergy, IWK Health Centre, Halifax, NS Canada; Western University, London, ON Canada; McMaster University, Hamilton, ON Canada

**Keywords:** Epinephrine, Anaphylaxis, Infant, CSACI position statement, Allergy

## Abstract

Epinephrine (adrenaline) is the treatment of choice for anaphylaxis. While other medications, including H_1_-antihistamines, H_2_-antihistamines, corticosteroids, and inhaled beta-2 agonists are often used to treat anaphylaxis in the emergency setting, none of these medications has been shown to reverse anaphylaxis. Fatal anaphylaxis is related to the delayed use of epinephrine. In community settings, epinephrine is available as an auto-injector in two doses, 0.15 mg and 0.3 mg. The recommended dose for children is 0.01 mg per kilogram. For infants at risk of anaphylaxis in the community, there are few options with regard to providing an optimal epinephrine dose for first-aid treatment. The Canadian Society of Allergy and Immunology (CSACI) therefore recommends, for the child weighing less than 15 kg, given the lack of a suitable alternative, prescribing the 0.15 mg epinephrine autoinjector. Adverse effects of an epinephrine dose of 0.15 mg given intramuscularly in infants or children weighing less than 15 kg are expected to be mild and transient at the plasma epinephrine concentrations achieved; therefore, these effects need to be measured against the consequences of not receiving epinephrine at all, which can include fatality.

Epinephrine (adrenaline) is the treatment of choice for anaphylaxis [1–3]. No other medication decreases airway edema (via its alpha-1 adrenergic effects), acts as a vasoconstrictor and relieves shock (via its alpha-1 adrenergic effects), has inotropic and chronotropic effects (via its beta-1 adrenergic effects), leads to bronchodilation (via its beta-2 adrenergic effects), and decreases mediator release (through mast cell stabilization) [1]. While other medications, including H_1_-antihistamines, H_2_-antihistamines, corticosteroids, and inhaled beta-2 agonists are often used to treat anaphylaxis in the emergency setting, none of these medications has been shown to reverse anaphylaxis [1]. While the same can be said regarding epinephrine, as there are no randomized double-blind controlled trials comparing epinephrine to placebo, robust epidemiological data including studies of fatal anaphylaxis, mechanistic data and animal studies, and years of clinical experience confirm that prompt injection of epinephrine is the best treatment of life-threatening anaphylaxis. Scrutiny of data from anaphylaxis fatality studies suggest that prompt injection of epinephrine is the best treatment of life-threatening anaphylaxis [4].

As outlined in the 2011 World Allergy Organization Guidelines, management of anaphylaxis includes the following instructions: 1) Have a written Emergency Action Plan; 2) Remove exposure to the trigger, if relevant/possible; 3) Assess the person’s reaction; 4) Call for help; 5) Inject epinephrine IM in the mid-outer aspect of the thigh; 6) Place the child on the back or in a position of comfort if there is respiratory distress and/or vomiting; elevate the lower extremities [1].

In community settings, epinephrine is available as an auto-injector in two doses, 0.15–0.3 mg. The recommended dose for children is 0.01 mg per kilogram, which can be repeated every 5–15 min as clinically indicated [1]. The Canadian Paediatric Society recommends using an epinephrine autoinjector 0.15 mg for children who weigh between 10 and 25 kg [2]. EpiPen® and Allerject® product monographs suggest that EpiPen®/Allerject® 0.15 mg should be given for children weighing between 15 and 30 kg and for those weighing less than 15 kg, 911 should be called [5, 6]. For infants at risk of anaphylaxis in the community, there are few options with regard to providing an optimal epinephrine dose for first-aid treatment [2, 7].

In obese or overweight children, there is some concern that the needle length may not be sufficient to penetrate the subcutanous tissue and reach the muscle. Stetcher *et al.* found that the needle lengths were not long enough to reach the muscle in a significant number of children [8]. In infants, the opposite is a concern: is the needle too long [9]? The hypothetical risks need to be balanced with the clear potential benefits.

Most anaphylaxis in infants occurs in community settings where cow’s milk is the most common trigger [10]. For outpatient use, the recommended epinephrine dose of 0.01 mg per kg is not currently available in auto-injector form less than 0.15 mg. In order to meet these dosing recommendations in smaller children, some physicians prescribe ampules of epinephrine, and parents have been instructed to draw up and administer epinephrine using ampules and syringes. This method may lead to inaccurate dosing and delays in administration [11]. A more acceptable solution, in the absence of a lower dose of epinephrine auto-injector, would be to prescribe the 0.15 mg epinephrine auto-injector dose [11–13].

This position statement will address a number of questions regarding epinephrine administration/prescribing suggestions for the infant under 15 kg who is at risk for anaphylaxis. It specifically addresses the following questions: What are possible consequences of administering a larger than recommended dose of epinephrine? Are there other ways to prescribe the recommended dose of epinephrine? What are the consequences of not administering epinephrine? What does the Canadian Society for Allergy and Clinical Immunology suggest for the infant less than 15 kg?

1. What are possible consequences of an epinephrine overdose?
  During a randomized, double-blind, parallel-group study, children age 4–8 years weighing 15–30 kg, and at risk of anaphylaxis in the community, self-injected either 0.15 mg or 0.3 mg of epinephrine. Transient dose-related adverse effects were observed after these injections. All children receiving the 0.15 mg dose developed pallor, and some also experienced tremor and anxiety. All children receiving the 0.3 mg dose experienced pallor, tremor, anxiety, and palpitations, and two children developed headache and nausea. One child who weighed 30 kg and received 0.3 mg epinephrine dose developed transient prolongation of the QTc interval [14]. Serious adverse events have been described in adult patients with anaphylaxis who received overdoses of IV epinephrine (cardiac resuscitation doses of 1 mg of 1:10 000 IV push bolus were given), or when rapid IV infusions were given [15, 16].
2. Are there other ways to prescribe the recommended dose of epinephrine?
  Given that currently the only epinephrine doses available in auto-injectors are 0.15 mg or 0.3 mg, another option is to prescribe 1 mL ampoules of epinephrine and 1 mL syringes and instruct parents how to draw up the prescribed dose for treatment of an anaphylaxis episode. One study specifically evaluated the ability of parents, physicians and nurses to quickly, and correctly draw up epinephrine via a syringe and ampoule. The results indicated that even in a calm setting, compared to the healthcare professionals, parents took significantly longer to draw up the dose, and the dose ultimately drawn up by parents had a 40-fold range of epinephrine content. Other concerns identified included difficulty removing air from the syringe without ejecting the epinephrine dose from the syringe, and in one instance a parent shattering the ampoule. Given that the goal of prescribing or providing a syringe and ampoule is to be more precise about epinephrine dosing, this is not an adequate solution [11].
3. What are the consequences of not administering epinephrine to infants weighing less than 15 kg?
  Severe, biphasic and fatal anaphylaxis have been reported in infants as young 7 weeks of age, highlighting that fatal anaphylaxis can occur in infancy [17]. Other studies have suggested that delay in administration of epinephrine can lead to more serious outcomes [18, 19]. The use of epinephrine is associated with lower hospitalization rates and reduced mortality [20]. Early recognition of anaphylaxis and prompt use of epinephrine can be particularly challenging in infants, who cannot communicate their symptoms and do not always develop hives or other obvious cutaneous signs of anaphylaxis [21].
4. What does the Canadian Society for Allergy and Clinical Immunology suggest for the infant less than 15 kg?
  The potential consequences of not administering epinephrine to a child with anaphylaxis outweigh the potential consequences of administering higher than recommended doses of epinephrine. Given the lack of suitable alternatives, the CSACI suggests that an epinephrine autoinjector of 0.15 mg be prescribed for children weighing less than 15 kg (including less than 10 kg) (expert opinion). Ideally, epinephrine auto-injectors containing a lower epinephrine dose, for example 0.1 mg, would be recommended for use in this population.

## Conclusion

For the child weighing less than 15 kg, given the lack of a suitable alternative, we recommend prescribing the 0.15 mg epinephrine autoinjector. Adverse effects of an epinephrine dose of 0.15 mg given intramuscularly in infants or children weighing less than 15 kg are expected to be mild and transient at the plasma epinephrine concentrations achieved; therefore, these effects need to be measured against the consequences of not receiving epinephrine at all, which can include fatality. The majority of physicians now prescribe an epinephrine auto-injector 0.15 mg for infants and children weighing less than 15 kg, in the absence of a weight-appropriate alternative (i.e., an epinephrine auto-injector containing lower doses). We discourage the prescription of epinephrine ampoules and syringes. Fatal and near-fatal outcomes are related to delayed administration of epinephrine, which should be used promptly, as it is the only medication known to reverse the life-threatening effects of anaphylaxis.

### Key points

- Epinephrine is the best medication to reverse anaphylaxis.
- Fatal anaphylaxis is related to the delayed use of epinephrine.
- It is essential to teach caregivers how to recognize anaphylaxis and promptly and correctly use the epinephrine auto-injector prescribed for the infant.
- We suggest prescribing a 0.15 mg epinephrine auto-injector for a child with a history of anaphylaxis weighing less than 15 kg, given the potential serious consequences of anaphylaxis, and the potential but generally mild adverse effects of epinephrine,.
- Ideally, epinephrine auto-injectors containing a lower epinephrine dose, for example 0.1 mg, would be recommended for use in this population.

## Abbreviations

CSACI: Canadian Society for Allergy and Clinical Immunology
IM: Intramuscular
IV: Intravenous
kg: Kilogram
mg: Milligram
